# Transoral Robotic Surgery in Retrostyloid Parapharyngeal Space Schwannomas

**DOI:** 10.1155/2014/296025

**Published:** 2014-08-18

**Authors:** Mohssen Ansarin, Marta Tagliabue, Francesco Chu, Stefano Zorzi, Michele Proh, Lorenzo Preda

**Affiliations:** ^1^Division of Head and Neck Surgery and Otorhinolaryngology, European Institute of Oncology, Via Ripamonti 435, 20141 Milan, Italy; ^2^Department of Otorhinolaryngology, IRCCS Policlinic San Matteo, Piazzale Golgi 2, 27100 Pavia, Italy; ^3^Division of Radiology, European Institute of Oncology, Via Ripamonti 435, 20141 Milan, Italy

## Abstract

Parapharyngeal space (PPS) tumors are very rare, representing about 0.5% of head and neck neoplasms. An external surgical approach is mainly used. Several recent papers show how transoral robotic surgery (TORS) excision could be a prospective tool to remove mainly benign lesions in PPS; no cases of neurogenic tumors from the retrostyloid space treated with TORS have been reported. We present two cases which underwent TORS for schwannomas from the retrostyloid compartment of the parapharyngeal space. Clinical diagnosis of schwannoma was performed by magnetic resonance imaging (MRI). In the first case a 6 cm neurogenic tumor arose from the vagus nerve and in the second case a 5 cm mass from the sympathetic chain was observed. Both cases were treated successfully by the TORS approach using a new “J”-shaped incision through the mucosa and superior pharyngeal constrictor muscle. Left vocal cord palsy and the Claude Bernard Horner syndrome, respectively, were observed as expected postsurgical sequelae. In case 1 the first bite syndrome developed after three months, while no complications were observed in case 2. Both patients regained a normal swallowing function. TORS seems to be a feasible mini-invasive procedure for benign PPS masses including masses in the poststyloid space.

## 1. Introduction

Parapharyngeal space (PPS) tumors represent 0.5% of head and neck neoplasms [1, 2]. The PPS is described as an inverted pyramid whose base is at the skull base level and apex at the hyoid bone. It is divided by Riolan's bundle (common tendon of the stylohyoid, styloglossus, and stylopharyngeal muscles) into the prestyloid or masseteric space and the retrostyloid or carotid artery space. The prestyloid compartment contains the deep lobe of the parotid gland, the internal maxillary artery, the ascending pharyngeal artery, fat, and lymph nodes. The retrostyloid space contains the internal carotid artery, the internal jugular vein, cranial nerves IX to XII, and the sympathetic chain [3, 4].

Schwannoma is the most common tumor of the PPS, arising more frequently from the cervical sympathetic chain or from the last four pairs of cranial nerves of the retrostyloid compartment [4–6].

About 80% of schwannoma cases are diagnosed by magnetic resonance imaging (MRI). MRI is the most sensitive and specific tool for identifying the origin of PPS masses [7, 8].

Different surgical approaches for PPS neoplasms have been reported (transoral, transcervical, with transcervical including parotidectomy or mandibulotomy) [3, 9].

Recently, TORS has emerged as a prospective tool to remove benign and small-sized lesions in PPS in the light of the restricted working space and the risk of injuries to the structures of the retrostyloid compartment. To our knowledge no author has ever applied TORS for the excision of neurogenic tumors arising from the retrostyloid compartment.

In this report we present two schwannomas of the retrostyloid compartment excised with TORS using a new “J”-shaped incision through the mucosa and superior pharyngeal constrictor muscle to obtain a wider vision of the structures of the upper portion of the PPS.

## 2. Case Presentation

We present two consecutive patients affected by schwannoma of the retrostyloid compartment of PPS diagnosed with MRI. Patients underwent TORS at the Head and Neck Division of the European Institute of Oncology, Milan.

### 2.1. Patient 1

A 39-year-old man, affected by vertigo, had undergone an MRI scan with an incidental finding of an unknown mass arising from the left parapharyngeal space. No symptoms were reported.

Oral examination showed a soft and painless bulge on the left posterior tonsillar pillar. The overlying mucosa was smooth and undamaged. On laryngoscopy, the mass extended downwards into the hypopharynx without involving the larynx. Its inferior edge was palpated just below the left angle of the mandible and presented as firm and nonfixed to the surrounding tissues.

**Figure 1:**
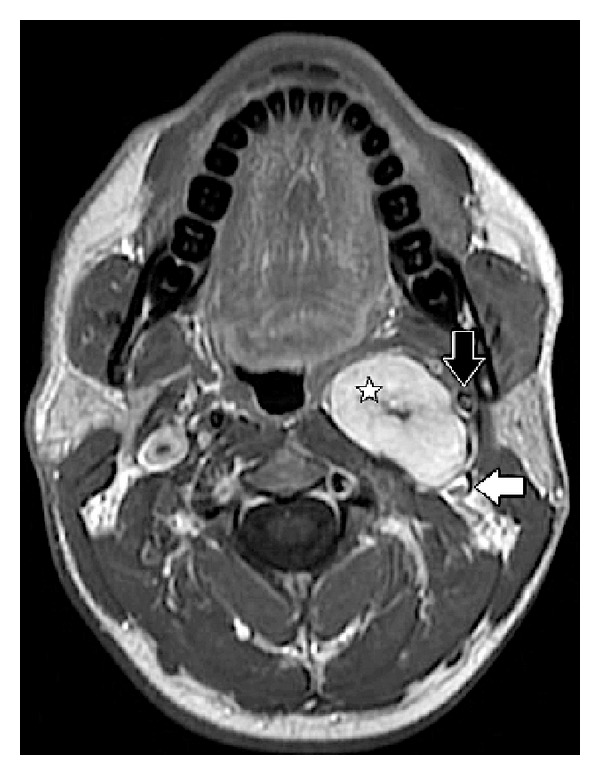
Patient 1 T1-weighted enhanced MRI scan: encapsulated tumor of the left retrostyloid compartment, 5 cm by 4 cm in size (white star). The internal carotid artery (black arrow) is displaced anterolaterally; the internal jugular vein is displaced posteriorly (horizontal white arrow).

Patient 1 referred to our institute with a neck ultrasound which showed an oval, hypoanechoic mass in the left parapharyngeal space, with peripheral vascular spots at US-Doppler evaluation. Ultrasound-guided fine-needle aspiration cytology (FNAC) was not diagnostic. Preoperative MRI scan showed a 48 × 39 × 25 mm well-defined mass located in the left retrostyloid PPS. The internal carotid artery (ICA) and external carotid artery were both displaced anterolaterally while the internal jugular vein (IJV) resulted displaced posterolaterally (Figure 1).

### 2.2. Patient 2

A 45-year-old woman presented a left submandibular swelling persisting for the last three years. Three years previously, the patient had undergone an ultrasound-guided FNAC, whose diagnosis was suggestive of a benign neurogenic tumor. She underwent an MRI examination which confirmed the presence of a 29 × 20 × 19 mm ovoid mass occupying the left retrostyloid PPS.

She refused surgery and decided with her specialist, upon a radiological followup, to undergo an MRI scan every year.

When the patient first referred to our clinic, the lesion had grown by approximately 5 mm. On physical examination the swelling was firm, mobile, nontender, and painless.

On videolaryngoscopy the hypopharynx and larynx were normal.

**Figure 2:**
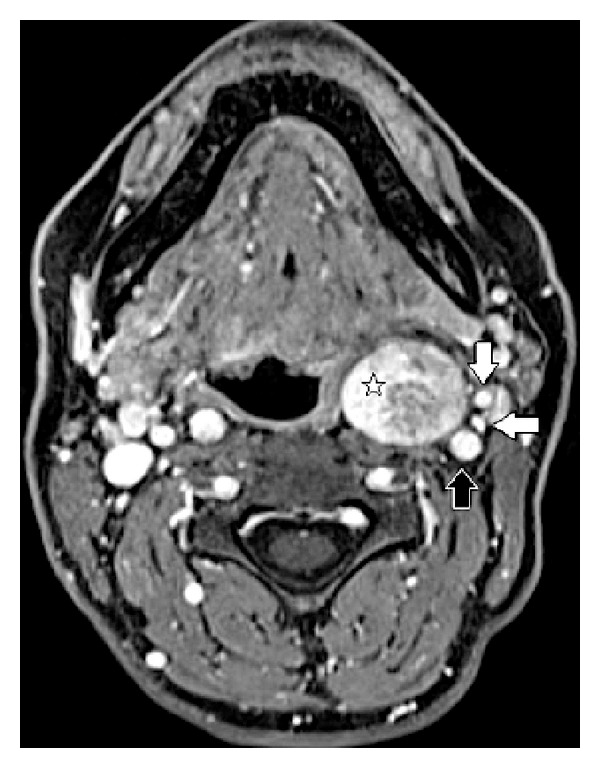
Patient 2, T1-weighted enhanced MRI scan: ovoid mass occupying the left retrostyloid compartment, 3 × 4 cm in size (white star). The internal carotid artery (black arrow), the internal jugular vein (horizontal white arrow), and the external carotid artery (vertical white arrow) are all displaced laterally.

The last MRI scan showed an ovoid mass occupying the left parapharyngeal space, 25 × 23 × 35 mm in size (versus 20 × 19 × 29 mm in 2010), with homogeneous contrast enhancement, low signal intensity on T1-weighted imaging, while it demonstrated increased T2-weighted signal intensity. The IJV and the ICA artery were both laterally displaced (Figure 2).

Both patients underwent preoperative MRI which showed sharply circumscribed ovoid masses with smooth margins, located in the retrostyloid PPS (Figures 1 and 2).

Both lesions showed a low signal on T1-weighted sequences, a high signal on T2-weighted sequences, and intense contrast enhancement, uniform in patient 1, slightly inhomogeneous in patient 2, on T1-weighted sequences after gadolinium injection.

The absence of high-velocity flow voids inside the lesions helped in the differential diagnosis with paragagliomas in both cases.

In patient 1 the lesion displaced the ICA in the anterolateral direction and the JV in the posterolateral direction resulting in the separation of the two vessels. Its upper pole was adjacent to the jugular foramen (Figure 1).

In patient 2 the lesion displaced both the ICA and the JV in the lateral direction with no significant separation between the two vessels (Figure 2).

According to the classification proposed by Furukawa et al. the supposed nerve of origin on the basis of MRI findings was the vagus nerve in patient 1 and the sympathetic chain in patient 2 [10].

### 2.3. Surgical Procedure

Patients underwent general anaesthesia via nasotracheal intubation. The Da Vinci system with three arms was used (one endoscopic arm with integrated cameras for the tridimensional view and two instrument arms, a 5 mm Maryland forceps and a 5 mm monopolar spatula cautery, interchangeable during the procedure). A bedside assistant was ready to suction and cauterise in case of intraoperative bleeding.

Transoral exposure was obtained with a Feyh-Kastenbauer retractor (Gyrus ACMI, Southborough, Massachusetts).

**Figure 3:**
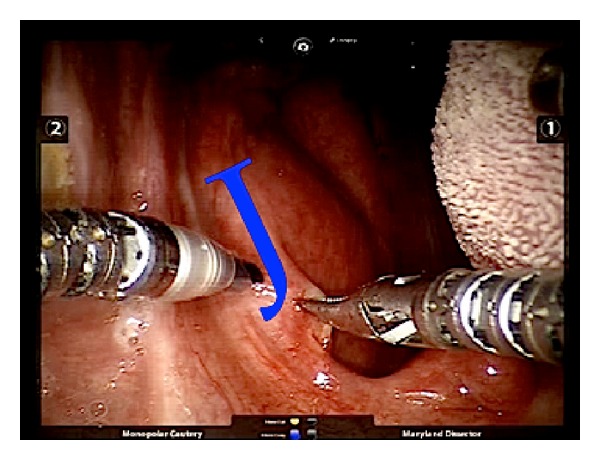
“J”-shaped incision on the anterior tonsillar pillar in order to obtain a wider and more detailed vision of the structures included in the upper portion of the PPS.

A 4 cm “J”-shaped incision through the mucosa and superior pharyngeal constrictor muscle was made using the monopolar cautery. Instead of performing the usual vertical incision, we decided to introduce this new modified “J”-shaped incision, to obtain a wider vision of the upper portion of the PPS (Figure 3).

**Figure 4:**
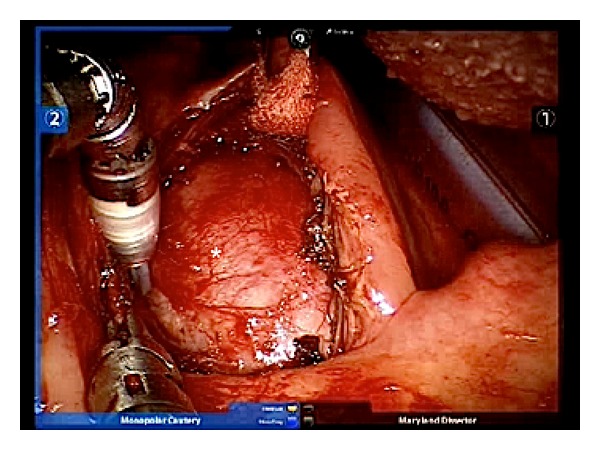
Retrostyloid schwannoma covered by surrounding fascia (white star).

After the incision of the mucosa, superior constrictor muscle, and pharyngobasilar fascia the tumour appeared as well-capsuled, hard, and elastic in consistency. The ICA had been anterolaterally displaced in both cases. The mass was gently detached from the surrounding fascia, preserving the capsule and separating it carefully from the ICA, with a spatula-tipped arm (Figure 4).

In both cases the nerve of origin was identified and resected. The masses were removed entirely without capsular rupture.

A thin haemostatic patch was positioned over the ICA. The surgical wound in both patients was sutured with absorbable suture.

In patient 1 a tracheotomy was performed to prevent blood inhalation in case of postoperative bleeding and removed on the third postoperative day, while in patient 2 it was not considered necessary.

The mean operative times for patient 1 and patient 2 were 254 min and 120 min, respectively. The time of hospitalization was 8 days for patient 1 and 7 days for patient 2. No nasogastric tube was needed. Liquid diet was possible from the day after surgery.

**Figure 5:**
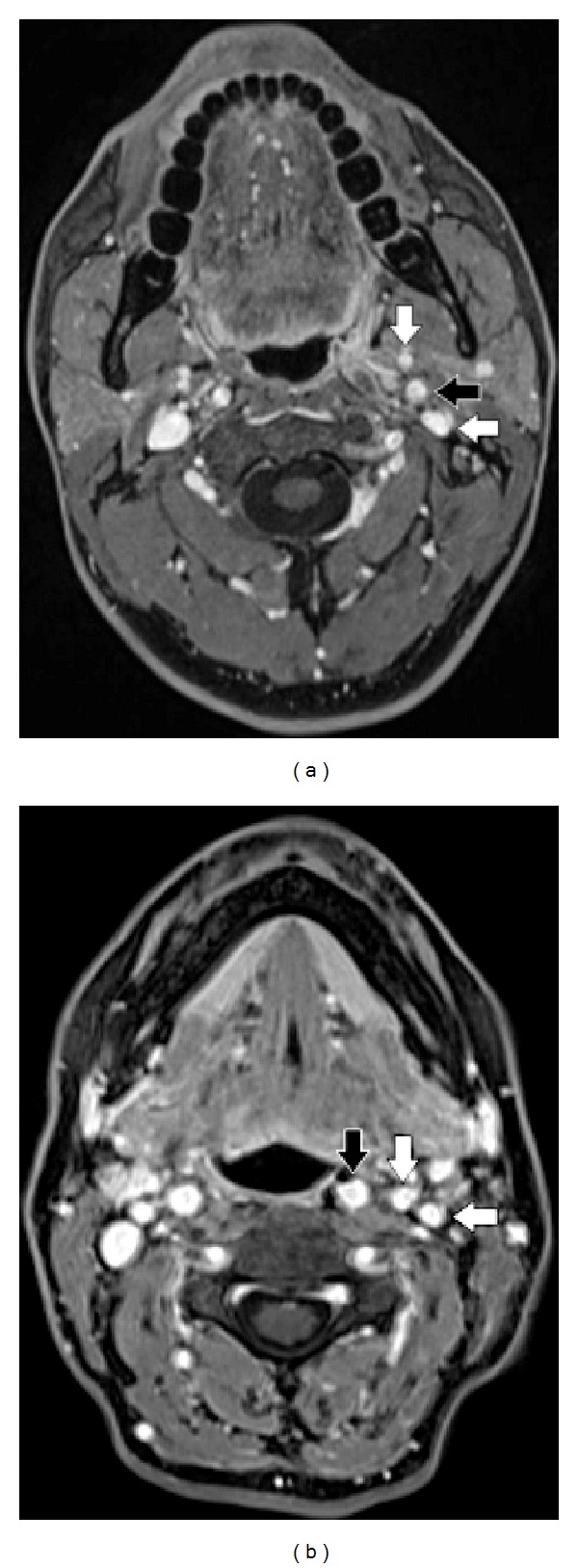
Patient 1 and patient 2, respectively: T1 weighted enhanced MRI scan. The internal carotid artery (black arrow), the external carotid artery (vertical white arrow), and the internal jugular vein (horizontal white arrow) are replaced in their original position with no evidence of disease.

The followup time was 7 months for patient 1 and 5 months for patient 2. Both patients are satisfied with no external visible scars and free from recurrences (Figures 5(a) and 5(b)).

As expected, patient 1 developed paralysis of the left vocal cord and patient 2 developed Horner's syndrome.

The histological examination confirmed the diagnosis of schwannoma in each patient, originating from the left vagus nerve and the left sympathetic chain, respectively. After three months patient 1 developed first bite syndrome (acute pain in the left parotid region after the first bite of each meal) [11] while in patient 2 Horner's syndrome improved.

## 3. Discussion 

Surgical excision is the treatment of choice for schwannomas of PPS. Slow growth and the noninvasive nature of schwannomas also allow an observational approach [7]. Treatment choice should be based on the surgical risk/benefit balance, that is, the severity of preoperative symptomatology and the anticipated postoperative neurological deficit.

An accurate preoperative identification of the nerve of origin, therefore, allows patients to make an informed decision on whether to undergo operation or observation [12].

The differential diagnosis must consider salivary tumors, metastatic lymph nodes, or soft tissue neoplasms: paragangliomas, aneurysms, branchial cleft cyst, or neurogenic tumors [2].

The surgical approach must guarantee a wide view of the surgical bed for a radical dissection of the tumor with the lowest risk of injuries to the vital structures and with minimal functional/cosmetic impairment [9, 13].

The “TORS in PPS” debate deals with its limited exposure of the tumor and the risk of tumor spillage/fragmentation. O'Malley et al. reported a successful excision of a benign salivary cyst of the PPS and 10 benign salivary neoplasms excised with TORS [14, 15]. Controversially, they reported one case which was converted to an open approach in order to preserve the ICA and three cases with tumor fragmentation/capsule disruption. For this reason, TORS is generally limited to selected tumors [16].

Ducic et al. reported a transoral approach to the poststyloid compartment concluding that TORS may not provide a full intraoperative control of lesions behind the ICA [17]. Conversely, O'Malley et al. described how a poststyloid tumor is not a contraindication for TORS [14, 15].

Two cases of PPS schwannomas excised with TORS alone have been reported, but none of them arose from the poststyloid compartment. It is yet to be verified whether TORS would be suitable for neurogenic tumors of the poststyloid compartment [18, 19].

To our knowledge this is the first paper reporting the safety and feasibility of TORS applied to neurogenic tumors arising from the poststyloid compartment.

In our series both tumors were radically removed and patients expressed complete satisfaction with their cosmetic and functional results. Patients 1 and 2 presented a left vocal cord palsy and Claude Bernard Horner syndrome, respectively.

Postsurgical sequelae were expected due to the resection of the X cranial nerve and cervical sympathetic trunk, performed in order to achieve a radical excision of the tumor. These neurologic symptoms are also reported with external approaches and as Lee et al. reported the absence of any neurologic deficit can be related only to schwannomas arising from the prestyloid compartment and originating from peripheral trigeminal branches [18].

Patient 1 also reported “first bite syndrome.”

First bite syndrome is described as a sudden onset of intense head and neck pain in the parotid region, sometimes extended to the homolateral neck and ear, with no prodromal symptoms triggered by the first bite and then gradually disappears. It is caused by sympathetic-parasympathetic conflict on the myoepithelial cells of the parotid region; in 95% of cases it follows upper neck surgery [11].

We speculate that in our case the first bite syndrome was subsequent to the loss of sympathetic innervation caused by an injury to the cervical sympathetic trunk. A vertical incision on the anterior tonsillar pillar through the superior pharyngeal constrictor muscle is usually reported [13, 20]. Instead of a vertical incision, we performed a “J”-shaped incision to obtain a wider vision of the structures of the upper portion of the PPS (Figure 3).

Our experience confirmed that TORS may overcome the limitations of visual and instrumental access of the classical transoral approach. The vital structures can be magnified and easily preserved. This technique permitted a clear identification of the tumor capsule and its delicate dissection. The 360-degree motion of the robotic system provided excellent manipulation of tissues to facilitate dissection, especially in the lateral plane [17].

For this reason, TORS is a feasible procedure for neurogenic tumors of the poststyloid compartment. A “J”-shaped incision on the anterior tonsillar pillar allows a wider and more detailed three-dimensional view of the structures in the upper portion of the PPS. Morbidity due to injuries to the neurovascular structures appears to be acceptably similar to external approaches but TORS does enable functional and cosmetic impairments to be avoided and carries a lower risk of adverse wound-healing events.
